# Complete Genome Sequence of an Extremely Halophilic Archaeon from Great Salt Lake, *Halobacterium* sp. GSL-19

**DOI:** 10.1128/MRA.00520-21

**Published:** 2021-07-15

**Authors:** Priya DasSarma, Brian P. Anton, Hedvig A. L. von Ehrenheim, Richard J. Roberts, Shiladitya DasSarma

**Affiliations:** a Institute of Marine and Environmental Technology, University System of Maryland, Baltimore, Maryland, USA; b Department of Microbiology and Immunology, University of Maryland School of Medicine, Baltimore, Maryland, USA; c New England BioLabs, Ipswich, Massachusetts, USA; d Blue Marble Space Institute of Science, Seattle, Washington, USA; Portland State University

## Abstract

An extremely halophilic archaeon, *Halobacterium* sp. GSL-19, was isolated from the north arm of Great Salt Lake in Utah. Single-molecule real-time (SMRT) sequencing was used to establish a GC-rich 2.3-Mbp genome composed of a circular chromosome and 2 plasmids, with 2,367 predicted genes, including 1 encoding a CTAG-methylase widely distributed among *Haloarchaea*.

## ANNOUNCEMENT

Halophilic microbes capable of surviving conditions with multiple extremes are of interest for biotechnology and astrobiology (1–8). To increase our understanding of these novel microbes, an extremely halophilic archaeon, GSL-19, was isolated from brine near the shore of the north arm of the Great Salt Lake in Utah (41.4377°N, 112.6689°W), proximal to the Spiral Jetty (9).

Brine was sampled from 10 cm below the surface of the lake at 28°C, inoculated into CM^+^ medium (complete medium plus trace elements), and grown with shaking at 220 rpm at 37°C, as previously described (10, 11). The enrichment cultures were plated on CM^+^ agar plates, and the isolate, an extremely halophilic, pigmented, phase-bright haloarchaeon, was purified by 3 rounds of streaking.

Nucleic acids were extracted using standard methods for haloarchaea involving hypotonic lysis phenol extraction and ethanol precipitation, as previously described (10–12), and sequencing was performed using the PacBio Sequel platform (Pacific Biosciences, Menlo Park, CA). SMRTbell libraries were prepared from 2 µg genomic DNA sheared to 40-kbp with a Megaruptor instrument (Diagenode, Inc., Denville, NJ), New England BioLabs (NEB) reagents equivalent to the PacBio library prep kit were used (13), and the library was sequenced on a single-molecule real-time (SMRT) cell with Sequel binding kit 3.0 with 10-h collection and 2-h pre-extension times. A total of 613,574 reads were obtained (subread *N*_50_, 4,078 bp), which were filtered and assembled *de novo* using Hierarchical Genome Assembly Process version 4 (HGAP4) with default parameters. The final assembly comprised 3 contigs, of which all circularized automatically using HGAP4, with mean coverage of 3,924×.

The genome (overall GC content of 66.7%) comprises a circular chromosome (1,987,132 bp, GC content of 68%) and 2 plasmids, namely, pGSL19_284 (284,178 bp, GC content of 59.1%) and pGSL19_54.9 (54,914 bp, GC content of 61.4%). Genes were predicted in-house using GeneMark HMM (14) and analyzed with HaloWeb (https://halo.umbc.edu), tRNAscan-SE2.0, and EMBOSS version 6.6.0.0 (15–17). The genome was also deposited at NCBI, where it was independently annotated using Prokaryotic Genome Annotation Pipeline (PGAP) Build 3190 (18).

The GSL-19 genome contained 2,367 genes, including a single rRNA operon and 44 tRNA genes all carried on the chromosome. The proteome was highly acidic (19–21), with a calculated mean pI value of 4.91 (22). All 799 core haloarchaeal orthologous groups (cHOGs) were encoded in the GSL-19 genome (23). The genome contained 8 genes encoding origin recognition complexes, 1 gene encoding a TATA-binding protein, and 5 genes encoding transcription factor B (24–26). A *gvp* gene cluster was also present, consistent with the production of gas vesicles observed as phase-bright inclusions (27, 28). Taxonomy was assigned using the 16S rRNA sequence and average nucleotide identity according to NCBI taxonomy, and the isolate has been designated *Halobacterium* sp. GSL-19 (29).

Methylated bases were determined using modification and motif analysis under the SMRTLink environment version 6.0.0.47841, revealing two methylated motifs, (m6A) GTCCAG (100%) and (m4C) CTAG (97.7%) (30). The CTAG methyltransferase gene is widely distributed among haloarchaea, in which CTAG sites are also underrepresented (31), suggesting a conserved function.

### Data availability.

The *Halobacterium* sp. GSL-19 genome sequence has been deposited in GenBank with the accession numbers CP070375.1 to CP070377.1, and raw data are available in the NCBI Sequence Read Archive with the accession number SRX10230949.
